# Distribution of NUDT15*4 in Native American populations of the Brazilian Amazon and risk of intolerance to thiopurine drugs

**DOI:** 10.3389/fphar.2026.1804744

**Published:** 2026-03-17

**Authors:** Guilherme Suarez-Kurtz, Anna Beatriz Ribeiro Elias, Marcelo Alex Carvaho, Alessandra Dias, Leonor Gusmão, Paulo Cesar Basta, Jamila Alessandra Perini

**Affiliations:** 1 Divisão de Pesquisa Clínica e Desenvolvimento Tecnológico, Instituto Nacional de Câncer (INCA), Rio de Janeiro, Brazil; 2 Instituto Federal do Rio de Janeiro (IFRJ), Rio de Janeiro, Brazil; 3 Laboratório de Diagnóstico por DNA (LDD), Universidade do Estado do Rio de Janeiro (UERJ), Rio de Janeiro, Brazil; 4 Departamento de Endemias Samuel Pessoa, Escola Nacional de Saúde Pública, Fundação Oswaldo Cruz (ENSP/Fiocruz), Rio de Janeiro, Brazil; 5 Laboratório de Pesquisa de Ciências Farmacêuticas (LAPESF), Universidade do Estado do Rio de Janeiro (UERJ), Rio de Janeiro, Brazil

**Keywords:** genetic polymorphism, indigenous people, native ancestry, personalized medicine, pharmacogenes, pharmacogenetic guideline, population diversity

## Abstract

**Introduction:**

Tolerance to thiopurine drugs is modulated by polymorphisms in the TPMT and NUDT15 pharmacogenes, affecting the activity of the encoded TPMT and NUDT15 enzymes. A recent study disclosed a significant impact of the NUDT15 rs147390019 single nucleotide variant (SNV) on thiopurine tolerance in acute lymphoblastic leukemia patients, self-identified as Hispanic/Latino. Native American (Amerindigenous) ancestry accounts almost exclusively for the presence of rs147390019 in Admixed Americans. We assessed the impact of rs147390019 (NUDT15*4) on the genotype-predicted risk of thiopurine intolerance in three Indigenous cohorts from reservation areas in the Brazilian Amazon, namely Munduruku (n = 77), Paiter-Suruí (n = 81) and Yanomami (n = 90).

**Methods:**

The individuals were genotyped using a panel of ancestry-informative markers and a validated TaqMan assay for the rs147390019 polymorphism.

**Results:**

The median proportion of Native ancestry in the cohorts was >99%. The rs147390019 SNP was not detected in Yanomami, but was present in Munduruku and Paiter-Suruí at the highest frequencies reported worldwide (0.130 and 0.191, respectively). Carriage of rs147390019 had a major effect on the distribution of inferred NUDT15 and combined NUDT15/TPMT metabolic phenotypes, such that the CPIC guideline recommendations for thiopurine dosing adjustments would apply to 29.9% and 70.4% of the Munduruku and Paiter-Suruí cohorts, respectively.

**Discussion:**

The results provide strong evidence to support inclusion of rs147390019 in pharmacogenetic testing panels to guide thiopurine dosing adjustments in Native and Latin American populations, acknowledging their ample genetic diversity and underrepresentation in pharmacogenetic research.

## 1 Introduction

Tolerance to thiopurine drugs, widely used in the treatment of lymphoid and myeloid leukemias, and non-malignant immunological syndromes, is modulated by polymorphisms in the *TPMT* and *NUDT15* pharmacogenes, affecting the activity of the encoded TPMT and NUDT15 enzymes. Accordingly, the Clinical Pharmacogenetic Implementation Consortium (CPIC; Maillard et al., 2026) and the Dutch Pharmacogenetics Working Group (DPWG; https://www.clinpgx.org/guidelineAnnotation/PA166184614) provide pharmacogenetic-guided thiopurine dosing algorithms based on genotype-inferred NUDT15 and TPMT metabolic phenotypes. Current testing for *NUDT15* risk variants focuses mainly on rs116855232, which is a single nucleotide variant (SNV; c.415C>T). This variant causes an arginine to be replaced by a cysteine at codon 139 (R139C), resulting in nearly complete loss of enzymatic activity and protein stability *in vitro*. The rs116855232 SNV defines the NUDT15*3 star allele and is also present in NUDT15*2; patients carrying these alleles are at increased risk of intolerance to thiopurines (Moriyama et al., 2016). A recent study in a large, multinational cohort of acute lymphoblastic leukemia (ALL) patients provided compelling evidence that another SNV affecting codon 139, namely rs147390019 (c.416G>A; R139H; NUDT15*4) increased the risk of intolerance to thiopurines to an extent similar to that observed for the rs116855232 SNV; of note, all carriers of rs147390019 self-identified as Hispanic/Latino (Maillard et al., 2025).

The peoples of Latin America are highly admixed, with major ancestral roots in the American continent, Europe and Africa. Local ancestry analyses displayed in the Genome Aggregation Database (gnomAD, https://gnomad.broadinstitute.org/) indicate that Amerindigenous (Native American, Amerindian) ancestry accounts almost exclusively for the presence of rs147390019 in Admixed Americans. Accordingly, this SNV is virtually absent in European and African groups of gnomAD and the 1000 Genomes Project (1KGP; https://www.ensembl.org/index.html). A previous study from our group assessed the distribution of the *NUDT15* rs116855232 SNV, as well as the most studied and clinically relevant *TPMT* SNVs in three distinct Indigenous groups living in reservation areas in the Brazilian Amazon, namely Munduruku, Paiter-Suruí and Yanomami (Perini et al., 2024). The results showed that the distribution of combined NUDT15/TPMT metabolic phenotypes differed largely across the Indigenous cohorts, and that the proportion of Paiter-Suruí individuals at risk of thiopurine intolerance (41% of the cohort) is 3- to 4-fold higher than any other population worldwide. Prompted by the recent study of Maillard et al. (2025) we assessed and now report the additional impact of the rs147390019 on the genotype-predicted risk of thiopurine intolerance in the Munduruku, Paiter-Surui, and Yanomami cohorts.

## 2 Materials and methods

This study is within the scope of a project approved by the Brazilian National Ethics Committee of Human Research (protocol number 65671517.1.0000.5240; Basta et al., 2021). The study participants, adult men and women, were recruited by a multidisciplinary team during their stay at three Indigenous reservation areas in the Brazilian Amazon, namely Sawré Muybu Indigenous land (Munduruku) between 29 October and 9 November 2019, Yanomami indigenous Land from the Ninam subgroup, between 4 and 14 October 2022 and Sete de Setembro Indigenous land (Paiter-Suruí) between 30 April and 5 May 2023. The location of the reservation areas and additional information on these Indigenous groups are presented in Perini et al. (2024). All participants provided written informed consent before enrollment; the leaders of the groups were apprised and granted approval for publication of the results of the project. All recruited Individuals have been previously genotyped with a panel of ancestry informative markers (Perini et al., 2025), and those with estimated proportions of Native ancestry >90% (77 Munduruku, 81 Paiter-Suruí and 90 Yanomami), were included in the present analysis.

A validated Taqman assay (Applied Biosystems, Foster City, California, United States) was used for allele discrimination of rs147390019 G>A (NUDT15*4); allele, genotype and diplotype frequencies were determined by gene counting. NUDT15, TPMT and combined NUDT15/TPMT metabolic phenotypes were assigned according to the updated Allele Functionality Tables in the CPIC thiopurine guideline, using data from the present study for rs147390019 and data from our previous study (Perini et al., 2024) in the same cohorts for *NUDT15* rs116855232, *TPMT* rs1142345, rs1800460 and rs1800462.

Chi-square tests were applied to assess deviations of genotype distribution from Hardy-Weinberg equilibrium (HWE) and to compare the distribution of alleles, genotypes and predicted metabolic phenotypes across the study cohorts. The Cramér´s V test (Cramér, 1946) was used to assess the strength (magnitude) of statistically significant associations in chi-square tests: the V values are interpreted according to Cohen´s h scale (Cohen, 1988). The FST statistics (Wright, 1978) was applied to characterize genetic differentiation (divergence) in rs147390019 across the Indigenous groups.

## 3 Results

Table 1 shows the estimated proportions of Native, European, and African ancestry, allele frequency and genotype distribution of *NUDT15* rs147390019 G>A in the study cohorts. Median proportion of Native ancestry exceeded 99% in the three cohorts. The rs147390019 SNV was absent in Yanomami but present in Munduruku and Paiter-Suruí, with minor allele frequency (MAF) of 0.130 and 0.191, respectively, and no deviation from HWE. The FST statistics indicated moderate genetic diversity among cohorts (FST = 0.07). The distribution of rs147390019 genotypes differed significantly (chi-square p < 0.0001) across the study cohorts; the Cramér´s V test pointed to medium magnitude of the effect (V = 0.28).

**TABLE 1:** Ancestry proportions, allele frequency, and genotype distribution of *NUDT15* rs147390019.

Cohorts	Munduruku (77)	Paiter-Surui (81)	Yanomami (90)
Biogeographical ancestry^a^			
Native American	99.3 (99.1–99.6)	99.5 (99.1–99.5)	99.3 (98.5–99.5)
African	0.2 (0.2–0.4)	0.3 (0.2–0.3)	0.3 (0.2–0.4)
European	0.4 (0.4–0.5)	0.3 (0.2–0.6)	0.5 (0.2–0.7)
*NUDT15* rs147390019^b^			
G	0.870	0.809	1.0
A	0.130	0.191	0
Fixation index *F*_*st*_ = 0.07			
G/G	0.753	0.630	1.0
G/A	0.234	0.358	0.0
A/A	0.013	0.012	0.0
Chi square *p* < 0.0001 Cramér V = 0.28			

^a^ Ancestry proportions expressed as median (interquartile range).

^b^ Allele and genotype frequency.

Data for *NUDT15* rs116855232, *TPMT* rs1142345, rs1800460 and rs1800462 from our previous study (Perini et al., 2024) were used in combination with the present results to assess the impact of rs147390019 on prediction of NUDT15 phenotypes and combined NUDT15/TPMT phenotypes in the study cohorts. The rs14730019 variant was absent in Yanomami, and consequently did not affect phenotype inference in this cohort. By contrast, the distribution of NUDT15 and combined NUDT15/TPMT phenotypes was markedly affected by rs14730019 in Paiter-Suruí and Munduruku (Table 2). Inclusion of rs147390019 in phenotype prediction led to (i) 5-fold and 25-fold increases in frequency of the NUDT15 intermediate phenotype (IM) in Paiter-Suruí and Munduruku, respectively; (ii) identification of one additional poor metabolizer (PM) in each cohort; and (iii) reduced prevalence of normal metabolizers (NM) in both cohorts. Regarding the combined NUDT15/TPMT phenotypes, taking rs14730019 into account led to: (i) 5.5-fold increase in number of IMs and identification of a PM among Munduruku; (ii) 3.5-fold increase in number of compound IMs (i.e; IMs for both NUDT15 and TPMT); (iii) identification of one more PM and 9 additional IMs among Paiter-Suruí.

**TABLE 2:** Distribution of predicted NUDT15 and combined NUDT15/TPMT metabolic phenotypes.

METABOLIC PHENOTYPES^a^	rs116855232	rs116855232 plus rs14730019	rs116855232	rs116855232 plus rs14730019
	Paiter-Suruí (81)		Munduruku (77)	
NUDT15				
Normal	0.88 (0.80–0.95)	0.49 (0.38–0.60)	0.99 (0.96–1.0)	0.74 (0.64–0.84)
Intermediate	0.09 (0.03–0.16)	0.47 (0.36–0.58)	0.01 (0–0.04)	0.25 (0.15–0.34)
Poor	0.03 (<0.01–0.09)	0.04 (<0.01–0.08)	0.0	0.01 (<0.01–0.04)
​	Chi square p < 0.0001	Cramér V = 0.40	Chi square p < 0.0001	Cramér V = 0.36
Combined NUDT15/TPMT				
Normal	0.54 (0.44–0.65)	0.30 (0.20–0.40)	0.95 (0.9–1.0)	0.70 (0.60–0.80)
Intermediate^b^	0.35 (0.24–0.45)	0.46 (0.35–0.57)	0.05 (0–0.1)	0.29 (0.18–0.38)
Poor	0.06 (<0.01–0.11)	0.07 (0.02–0.13)	0.0	0.01 (<0.01–0.04)
Compound intermediate^c^	0.05 (<0.01–0.10)	0.17 (0.09–0.26)	0.0	0.0
​	Chi square *p* = 0.005	Cramér V = 0.30	Chi square *p* = 0.003	Cramér V = 0.30

^a^ NUDT15 phenotypes predicted from rs116855232 alone (data from Perini et al., 2024) or from rs116855232 and rs147390019 (present study); TPMT phenotypes predicted from TPMT rs1142345, rs1800460 and rs1800462 (data from Perini et al., 2024); combined NUDT15/TPMT phenotypes according to CPIC, guidelines (Maillard et al., 2026). Data expressed as frequency (CI, 95%).

^b^ Intermediate metabolizer for either NUDT15 or TPMT.

^c^ Intermediate metabolizer for both NUDT15 and TPMT.

Collectively, these data indicate that the CPIC recommendations for thiopurine dosing adjustments in IMs, PMs, and compound IMs (Maillard et al., 2026), apply to 4.0%, 29.9% and 70.4% in the Yanomami, Munduruku, and Paiter-Suruí cohorts, respectively, when rs147390019 (NUDT15*4) was considered along with *NUDT15* rs116855232, *TPMT* rs1142345, rs1800460 and rs1800462 in inference of the combined NUDT15/TPMT metabolic phenotypes. Comparison with our precious data for the same cohorts (Perini et al., 2024), reveals that inclusion of rs147290019 in phenotype inference increases the proportion of individuals with CPIC thiopurine dosing recommendations from 5.2% to 29.9% in Munduruku and from 45.7% to 70.4% in Paiter-Suruí.

## 4 Discussion

We assessed the distribution of rs147390019G>A, which defines the NUDT15*4 star allele, in Native American cohorts recruited in three Indigenous reservation areas in the Brazilian Amazon. The variant allele was not detected in Yanomami, but was present in Munduruku (MAF 0.130) and Paiter-Suruí (MAF 0.191). Genetic drift, which prevails among Amerindian populations (Cavalli-Sforza et al., 1994; Salzano and Bortolini, 2002) may be invoked to account for the variable MAF of *NUDT15* rs147390019 across the study cohorts. Allelic fluctuations of another *NUDT15* SNV (rs116855232) and of polymorphisms in other pharmacogenes, notably *TPMT*, in distinct Native American peoples living in Brazil have been previously reported, and attributed to evolutionary processes, particularly genetic drift (Suarez-Kurtz et al., 2019; Perini et al., 2024).

In previous studies of Native Amerindian populations, the MAF of rs147390019 was reported at 0.016 in 64 individuals from 12 Amazonian groups (Rodrigues et al., 2020) and 0.10 in 50 samples from 5 Native groups of the Human Genome Diversity Project (HGDP; Cavalli-Sforza, 2005). In the HGDP Paiter-Suruí individuals (n = 7), the MAF of rs147390019 was 0.50: the small number of samples might account for the discrepancy with our findings for the Paiter-Suruí cohort (MAF 0.191), although the data point to the Paiter-Suruí group having the highest MAF of rs147390019 worldwide. Indeed, the MAF of rs147390019 in the 1KGP superpopulations ranges from zero to 0.009 (https://www.ensembl.org/index.html), while gnomAD (https://gnomad.broadinstitute.org/) shows global MAFs ranging from zero to 0.018. Of note, the MAFs of rs147390019 in Paiter-Suruí (0.191) and Munduruku (0.130) are considerably higher than the estimated MAF (0.064) for the Amerindigenous genetic ancestry of Admixed Americans in the gnomAD database. For comparison, in a large Brazilian cohort (SABE; Naslavsky et al., 2022) with negligible representation of Indigenous persons, the MAF of rs147390019 was reported at 0.005 (https://abraom.ib.usp.br/). Homozygosis for the variant rs147390019 allele, detected in one Paiter-Suruí and one Munduruku, has been previously reported in one HGDP Paiter-Suruí and 13 out of the nearly 60,000 gnomAD Admixed Americans, while no homozygosis was detected in all other gnomAD or HGDP groups, all 1 KG superpopulations, the SABE Brazilian cohort and 1,399 ALL multinational patients (Maillard et al., 2025).

The study of Maillard et al. (2025) provided compelling evidence that individuals heterozygous for the rs147390019 variant (NUDT15 *1/*4 genotype) may be considered as NUDT15 IM, and those with NUDT15*4 in homozygosity as NUDT15 PM. Accordingly, rs147390019 had a major impact on the inference of NUDT15 and combined NUDT15/TPMT metabolic phenotypes in Paiter-Suruí and Munduruku, such that the CPIC guideline recommendations for thiopurine dosing adjustments in IMs, PMs and compound IMs would apply to 29.9% and 70.4% of individuals in these cohorts, respectively. These proportions are, to the best of our knowledge, the highest reported worldwide.

The results of this study support the notions that (i) inclusion of rs147390019 (NUDT15*4) in genotype-prediction of NUDT15 phenotypes has “significant implication for clinical practice, especially for Hispanic/Latino patients” (Maillard et al., 2025) and (ii) *NUDT15* testing should become standard clinical practice before prescribing thiopurines in individuals with Amerindian/Alaska Native ancestry, including Hispanic individuals” (Ioannou et al., 2025). Accordingly, genotyping of rs147390019 at our institution revealed that 3 out 223 ALL Brazilian pediatric patients, previously classified as NUDT15 NMs had the NUDT15*1/*4 genotype, requiring re-classification as NUDT15 IMs (Suarez-Kurtz, unpublished observation).

A distinct feature of the present study is the enrollment of participants with individual proportions of Native ancestry >90%, based on estimates from a validated panel of ancestry informative markers (Perini et al., 2025). The median proportion of Native ancestry exceeded 99% in the three study cohorts, thus minimizing the potential influence of European and/or African admixture. Nevertheless, we acknowledge the relatively low number of participants (77–90) in the three cohorts as a limitation of our study, but note that practical and ethical difficulties are commonly encountered in recruiting participants from Native American populations, and that the number of participants from each cohort exceed largely those of previous studies of rs147390019 in Native American groups (Cavalli-Sforza, 2005; Rodrigues et al., 2020). Additional limitations of this study are: first, the cohorts investigated do not fully represent the diversity and heterogeneity of Native American peoples; indeed, the most recent Brazilian Census (2022) identified 391 distinct Amerindian ethnicities, speaking 295 languages (https://agenciadenoticias.ibge.gov.br/agencia-noticias/2012-agencia-de-noticias/noticias/44848-censo-2022-brasil-tem-391-etnias-e-295-linguas-indigenas); second, the inference of NUDT15 and compound NUDT15/TPMT metabolic phenotypes was based on genotyping of the most frequent and clinically characterized loss-of-function PGx variants listed in the CPIC guideline for thiopurines, and the reference allele for each gene was assigned by default.

In conclusion, this is the first report of the distribution of the rs147390019 SNV (NUDT15*4) in three distinct Native populations from Indigenous reservation areas in the Brazilian Amazon, namely Munduruku, Paiter-Suruí and Yanomami. All participants had estimated Native ancestry >90%. The rs147390019 MAF ranged from zero – 0.191 across cohorts, consistent with the ample genetic diversity of Native American peoples; the MAF in Munduruku (0.130) and Paiter-Suruí (0.191) are seemingly the largest reported worldwide. The presence of rs147390019 in Paiter-Suruí and Munduruku had major impact on genotype-prediction of NUDT15 and combined NUDT15/TPMT metabolic phenotypes and, consequently, on the proportion of individuals with CPIC guideline recommendations for thiopurine dosing adjustments. Collectively, the results of this study provide strong support for inclusion of rs147390019 in PGx panels to guide thiopurine dosing adjustments in populations with Native American ancestry.

## Data Availability

The datasets for this study can be found at https://doi.org/10.6084/m9.figshare.31315546.
